# American Ginseng Attenuates Eccentric Exercise-Induced Muscle Damage via the Modulation of Lipid Peroxidation and Inflammatory Adaptation in Males

**DOI:** 10.3390/nu14010078

**Published:** 2021-12-25

**Authors:** Ching-Hung Lin, Yi-An Lin, Shu-Li Chen, Mei-Chich Hsu, Cheng-Chen Hsu

**Affiliations:** 1 Physical Education Office, Yuan Ze University, Taoyuan 32003, Taiwan; lch0325@saturn.yzu.edu.tw; 2 Department of Sports Medicine, Kaohsiung Medical University, Kaohsiung 80708, Taiwan; vn508773@gmail.com; 3 Graduate Institute of Sports Science, National Taiwan Sport University, Taoyuan 33301, Taiwan; b506091076@tmu.edu.tw; 4 Department of Medical Research, Kaohsiung Medical University Hospital, Kaohsiung 80708, Taiwan; 5 Substance and Behavior Addiction Research Center, Kaohsiung Medical University, Kaohsiung 80708, Taiwan; 6 Department of Anatomy and Cell Biology, School of Medicine, College of Medicine, Taipei Medical University, Taipei City 11031, Taiwan

**Keywords:** antioxidants, cytokines, downhill running, ginsenoside, sports nutrition

## Abstract

Exercise-induced muscle damage (EIMD) is characterized by a reduction in functional performance, disruption of muscle structure, production of reactive oxygen species, and inflammatory reactions. Ginseng, along with its major bioactive component ginsenosides, has been widely employed in traditional Chinese medicine. The protective potential of American ginseng (AG) for eccentric EIMD remains unclear. Twelve physically active males (age: 22.4 ± 1.7 years; height: 175.1 ± 5.7 cm; weight: 70.8 ± 8.0 kg; peak oxygen consumption [$\dot{V}O_{2}$peak] 54.1 ± 4.3 mL/kg/min) were administrated by AG extract (1.6 g/day) or placebo (P) for 28 days and subsequently challenged by downhill (DH) running (−10% gradient and 60% $\dot{V}O_{2}$peak). The levels of circulating 8-iso-prostaglandin F 2α (PGF2α), creatine kinase (CK), interleukin (IL)-1β, IL-4, IL-10, and TNF-α, and the graphic pain rating scale (GPRS) were measured before and after supplementation and DH running. The results showed that the increases in plasma CK activity induced by DH running were eliminated by AG supplementation at 48 and 72 h after DH running. The level of plasma 8-iso-PGF2α was attenuated by AG supplementation immediately (*p* = 0.01 and *r* = 0.53), 2 h (*p* = 0.01 and *r* = 0.53) and 24 h (*p* = 0.028 and *r* = 0.45) after DH running compared with that by P supplementation. Moreover, our results showed an attenuation in the plasma IL-4 levels between AG and P supplementation before (*p* = 0.011 and *r* = 0.52) and 72 h (*p* = 0.028 and *r* = 0.45) following DH running. Our findings suggest that short-term supplementation with AG alleviates eccentric EIMD by decreasing lipid peroxidation and promoting inflammatory adaptation.

## 1. Introduction

Exercise-induced muscle damage (EIMD) and delayed onset muscle soreness (DOMS) are muscular adaptation processes that occur following exercise training. Prolonged and overloaded exercise training results in muscle fiber microdamage and increased oxidative stress. Thus, muscle soreness and function loss are frequently experienced during the recovery phase after exercise training [1]. Muscle repair occurs by regulating inflammatory activity and activating muscle stem cells (satellite cells) for cell repair and regeneration within injured muscle fibers [2]. Complete muscle repair may depend on rest, nutritional supplementation, and physical therapy. EIMD in professional athletes induces light or medium DMOS and affects muscle function within 2 to 3 days after exercise training. In contrast, the non-trained population may experience both severe DOMS and loss of muscle function due to EIMD; furthermore, a longer recovery phase is required in such cases for complete muscle repair [3,4].

Specific proteins and kinases in muscle fibers, such as creatine kinase (CK), lactate dehydrogenase (LDH), and myoglobin, are biomarkers of muscle damage. These resident proteins and kinases of muscle fibers enter circulation due to the alteration of membrane permeability caused by sarcolemma membrane injury [4]. Thus, the circulating levels and activities of CK, LDH, and myoglobin have been frequently researched to evaluate muscle damage. In addition to the intensity of the initial mechanical stress, the influence of oxidative stress and inflammatory reactions may play a critical role in muscle repair [5,6]. To respond to the heavy requirement of energy output during exercise, a large number of free radicals are generated and leaked from the electron transport chain accompanied by an accelerated metabolic rate, which eventually results in elevated levels of cellular lipid peroxidation and protein carbonylation products [5]. Antioxidant supplementation was shown to attenuate the elevation of oxidative stress and muscle damage following downhill (DH) running [7]. The regulation of oxidative stress during or following exercise training is associated with the extent of the inflammatory response [8,9,10].

The inflammatory response is an important process in physiological adaptation following exercise training. For muscle repair and regeneration, the inflammatory reaction activates cellular signaling for satellite cell proliferation and attracts immune cells for the removal of muscle fiber debris [11]. A previous study suggested that strength loss induced by muscle damage is associated with immune-cell infiltration [6]. Moreover, the activation and migration of immune cells are mediated by the secretion of cytokines and chemokines. Secretory changes in local and circulating cytokines have been widely applied to understand the progress of inflammation and repair following muscle damage [12]. Tumor necrosis factor (TNF)-α, interleukin (IL)-1β, and IL-6 are major pro-inflammatory cytokines involved in the initial stages of recovery [3]. Pro-inflammatory cytokines are secreted to attract specific immune cells to the necrotic site of muscle fibers and stimulate muscle regeneration [13]. In the late stages of recovery, the critical cytokines are anti-inflammatory cytokines, such as IL-4 and IL-10, which are responsible for the deactivation of active neutrophils and M1 macrophages [12,14]. Although the pro-inflammatory reaction is beneficial for triggering muscle generation, the prolonged high-level secretion of pro-inflammatory cytokines may exert negative effects on myocyte fusion and myogenic differentiation [13].

*Panax quinquefolium* L. (American ginseng, AG) is a popular herbal medicine that has been reported to possess a wide range of pharmacological properties in traditional Chinese medicine (TCM) [15]. AG displays pharmacological activities for the improvement of cognitive dysfunction, abnormalities of the immune system, cardiovascular diseases, diabetes, and cancers [15,16]. Ginsenosides are critical phytochemicals and major bioactive components of ginseng roots. AG differs from Asian ginseng (Panax ginseng) in its ginsenosides composition. AG has a higher level of total ginsenosides and abundant Rb1 and Re ginsenosides compared with Asian ginseng [16]. In a randomized, double-blind, placebo-controlled crossover study, 28-day supplementation of AG did not alter the running endurance but attenuated the plasma CK activity induced by exhaustive running exercise [17]. Moreover, ginsenosides Rb1 and Re show antioxidative activities, which have been demonstrated in both in vitro and in vivo studies [18,19,20,21].

Eccentric exercise is attracting increasing interest not only in sports science but also in clinical medicine because of its mechanical and metabolic properties [22]. Eccentric muscle contraction induces strong stimuli for working muscles; thus, for people with poor conditioning or strength, low-intensity eccentric training may provide similar benefits to traditional training [23]. However, eccentric exercise may result in EIMD and DOMS when participants are unaccustomed to eccentric exercise or untrained. To date, numerous studies have reported that various nutritional and supplementation strategies may efficiently attenuate EIMD and DOMS or improve the recovery rate following eccentric exercise [24]. Although AG has been widely investigated in many medical applications, there is no clear information on the protective effects of AG on eccentric EIMD.

Thus, in this study, we aimed to evaluate the effects of the short-term supplementation of AG on muscle damage, oxidative stress, and inflammation following acute DH running. We hypothesized that AG supplementation would attenuate an EIMD-induced increase in CK activity, muscle soreness, and lipid peroxidation and would also ameliorate pro-inflammatory responses and promote anti-inflammatory responses after DH running. The objectives of this study were (1) to identify the protective effects of AG supplementation on muscle damage by evaluating the changes in CK activity and DOMS, (2) to evaluate the anti-oxidative effects of AG supplementation on lipid peroxidation, and (3) to determine the changes in pro- and anti-inflammatory cytokines after DH running.

## 2. Materials and Methods

### 2.1. Subjects

**Figure 1:**
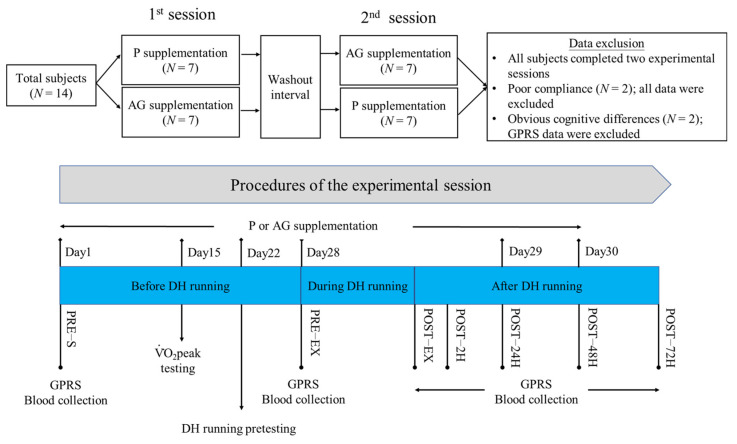
Experimental arrangement and procedure. AG: American ginseng; DH: downhill EX: exercise; GPRS: graphic pain rating scale; P: placebo.

Fourteen male physically active college students (age: 22.4 ± 1.7 years) were recruited for this randomized, double-blinded, crossover, placebo-controlled study. The average height, weight, and peak oxygen consumption ($\dot{V}O_{2}$peak) of the subjects were 175.1 ± 5.7, 70.8 ± 8.0 kg, and 54.1 ± 4.3 mL/kg/min (range from 47.1–59.8 mL/kg/min), respectively. Inclusion criteria required individuals to participate in moderate to vigorous exercise for at least 150 min or 3 times per week. Exclusion criteria included (1) regular use of dietary supplements or drugs, (2) history of cardiovascular, musculoskeletal, or immune system disorders, and (3) habits of smoking or alcohol consumption. As shown in Figure 1, the subjects were randomly assigned in a 1:1 ratio to one of the two subpopulations by drawing lots. After simple randomization, all subjects were allocated to experience two different experimental sessions in sequence. A supplement-free washout interval of more than 7 days was implemented between two experimental sessions. All subjects completed all experimental sessions. The measurements of two subjects were excluded because they did not comply with the requirements of this study (*N* = 12).

Most subjects entered the first experimental session approximately one month after the initial meeting. Except for data analysis, all subjects completed all experimental sessions between March and August. The subjects maintained their usual exercise habits and were asked not to use nutritional supplements or drugs during the experimental period. All participants were asked to maintain their dietary habits during the experimental period and to stop high-intensity exercise/physical activity for 3 days before blood sample collection. The contents, procedures, requirements, and possible risks of this study were clearly explained to each subject during their first recruitment visit. All participants provided written informed consent. This study was approved by the institutional review board of the National Taiwan Sports University (No. NCPESIRB-T951221-11) and was performed in compliance with the Declaration of Helsinki.

### 2.2. Experimental Procedures

This study followed a crossover study design and consisted of two experimental sessions for each subject. As shown in Figure 1, a 30-day supplementation of AG or placebo (P) was completed for each experimental session. To avoid any potential biological effects of supplementation on the $\dot{V}O_{2}$peak, $\dot{V}O_{2}$peak tests and DH running pretests were performed in sequence during the supplementation period. After completing the 28-day supplementation, DH running was performed to induce oxidative stress and muscle damage in each subject. Biochemistry markers and muscle soreness were evaluated before and within three days after DH running. Subjects were asked to stop any vigorous exercise/activity 3 days before DH running. On the 28th day of each session, subjects arrived at the laboratory between 7 and 9 a.m. following a 10-h fasting period. After completing the graphic pain rating scale (GPRS) and blood collection, each subject ingested either AG extract or hydroxymethylcellulose in the same capsule volume (4 × 400 mg). Subsequently, a 15-min warm-up activity was followed by a 60-min DH running (~60% $\dot{V}O_{2}$peak; −10% gradient). The average DH running speed among subjects was 10.1 ± 0.7 km/h (range between 48.2 and 60.8 mL/kg/min). The average temperature and humidity were 25.0 ± 1.2 °C and 58.4 ± 7.7%, respectively. Blood samples were collected, and GPRS was measured immediately and at 2, 24, 48, and 72 h after DH running. Within 72 h after DH running, no other nutritional supplements and drugs were used, and subjects were instructed to refrain from any exercise/activity.

### 2.3. Administration of AG Extract or Placebo

AG extract and hydroxymethylcellulose (placebo) were provided by Taiwan Biotech Co. (Taoyuan, Taiwan) and filled in the same capsule form (400 mg/capsule). The major bioactive ginsenosides present in the AG extract were Rb1 (8.67%, *w*/*w*), Rc (0.99%, *w*/*w*), Rd (1.05%, *w*/*w*), and Re (5.08%, *w*/*w*). As shown in Figure 1, subjects were instructed to ingest either AG capsules (4 × 400 mg AG extract) or placebo capsules (4 × 400 mg hydroxymethylcellulose) once a day in the morning during the experimental period. The washout period was more than seven days between the two sessions. These materials have been used in our previous study and have been proven to attenuate CK activity induced by submaximal exercise [17].

### 2.4. $\dot{V}O_{2}$peak Testing and 60% $\dot{V}O_{2}$peak DH Running

The exercise intensity used for DH running was referenced and modified according to previously published studies [13,24]. First, the subjects were tested on a treadmill at a 0% gradient (Vision Fitness HRC T8600, Lake Mills, WI, USA) to determine their $\dot{V}O_{2}$peak. The test protocol used to determine the $\dot{V}O_{2}$peak started at a treadmill velocity of 6 km/h, and then the treadmill speed was increased by 2 km/h every 3 min until exhaustion. Heart rate (HR; beats per minute, bpm) and rating of perceived exertion (RPE) were recorded using the Polar Electro PE300 sports tester (Kempele, Finland) and the Borg scale, respectively [25]. Both heart rate and RPE were recorded at the end of each minute. During the $\dot{V}O_{2}$peak test, continuous respiratory data were collected and analyzed using a cardiopulmonary exercise testing instrument (Vmax 29; Sensor Medics, Anaheim, CA, USA). At least two of the following criteria had to be attained for the determination of the $\dot{V}O_{2}$peak: (1) subjects were unable to keep up with the treadmill speed; (2) HR was within 10 bpm of the maximal predicted HR (220-age); (3) the RPE was greater than 19; and (4) the respiratory exchange ratio was greater than 1.1.

A DH running pretest was conducted to determine the treadmill speed used during DH running (Figure 1). The procedures of the DH running pretest were implemented as described below. The speed used in DH running was initially predicted using the linear regression equation obtained from data of running speed and the oxygen uptake value collected in the $\dot{V}O_{2}$peak testing. The predicted speed was applied to treadmill running with a −10% gradient (equal to −5.7 degrees) for each subject, and then the speed was adjusted to meet the exercise intensity at 60% $\dot{V}O_{2}$peak. The running test for determining the expected speed was completed within 20 min. Finally, the determined speed was applied to 60 min DH running with a −10% gradient. Before each exercise test or DH running, all subjects completed a 15-min warm-up activity.

### 2.5. GPRS

The GPRS test was used to evaluate muscle soreness [25]. The scale was a 12-cm straight line in the horizontal direction, from left to right, indicating seven different levels of pain, that is, no pain, dull pain, slight pain, increased mild pain, painful, very painful, and unbearable pain. The x mark indicating the extent of the subject’s muscle soreness was recorded by our staff. The distance between the x mark and the start was measured using a ruler as an indicator of muscle soreness. The time point when the GPRS test was performed is shown in Figure 1.

### 2.6. Blood Sample Collection

The time point of blood collection is shown in Figure 1. At each sampling point, approximately 20 mL venous blood was collected and added to a tube containing heparin or EDTA. Plasma separation was performed by a cooling centrifuge, and then the samples were stored at −80 °C for future analysis of biochemical markers.

### 2.7. Biochemical Measurements

Enzyme-linked immunosorbent assay (ELISA) kits for TNF-α, IL-1β, IL-4, and IL-10 were purchased from eBioscience (San Diego, CA, USA) and used to evaluate the levels of plasma inflammatory cytokines. The 8-iso-prostaglandin F 2α (PGF2α) ELISA kit (Cayman Chemicals, Ann Arbor, MI, USA) was used to determine the level of lipid peroxidation in the plasma. An Infinite M200 microplate reader (Tecan, Grödig, Austria) was used to determine the optical density value. A Johnson & Johnson DT-60II chemistry analyzer (Ortho Clinical Diagnostics, Rochester, NY, USA) was used to measure CK activity in the plasma. All assay kits were commercially available and operated according to the manufacturer’s instructions.

### 2.8. Statistical Analyses

The Shapiro–Wilk test was used to determine the normality of parameters. A nonparametric statistical method was used to evaluate the differences between AG and P supplementation because the data were non-normally distributed. The Friedman test and Wilcoxon signed-rank test were used to evaluate intergroup and intragroup differences. All data were displayed as median with interquartile ranges (IQR). Statistical significance was set at a *p*-value of less than 0.05. The effect size (ES) measurement used with Wilcoxon signed-rank test was performed using the *Z*-score to calculate the correlation coefficients using the below formula (where *N* was a total number of observations on which *Z* was based) [26]. The result of the ES measurement was presented as an *r*-value. According to Cohen’s guidelines, a large ES is *r* = 0.5, a medium ES is *r* = 0.3, and a small ES is *r* = 0.1 [27]. Power analysis was implemented using the G*Power software (version 3.1.9.4). The post hoc power analysis for Wilcoxon signed-rank test was applied to calculate the actual power value, and the input parameters were set at two-tailed, *α* = 0.05, sample size = 12, and determined ES.IBM SPSS statistical software (version 20.0) was used to perform all analyses.

$$\gamma =\frac{Z}{\sqrt{N}}$$

## 3. Results

### 3.1. Muscle Damage and Soreness

**Figure 2:**
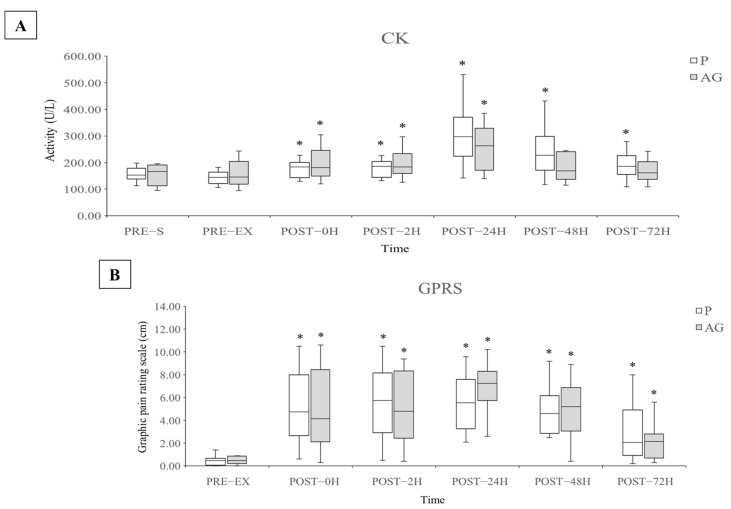
Levels of CK and muscle soreness affected by supplementation P and AG after DH running. Q1, median, Q3, minimum, and maximum values of CK (**A**) and GPRS (**B**) are presented in boxplots (*N* = 12 for CK; *N* = 10 for GPRS). * *p* < 0.05 vs. PRE-EX. AG: American ginseng; CK: creatine kinase; GPRS: graphic pain rating scale; P: placebo.

The CK activity and GPRS values were used to evaluate the EIMD and DOMS caused by DH running. As shown in Figure 2, the CK activity immediately increased and peaked 24 h after DH running. The average CK activity at POST-24H increased to 223% and 191% by supplementation P (*p* = 0.002 and *r* = 0.62) and AG (*p* = 0.003 and *r* = 0.61), respectively, compared with the PRE-EX level. The CK activity after the supplementation of both AG and P gradually decreased at 48 and 72 h after DH running. The CK activity returned to the pre-DH running level after AG supplementation and not after P supplementation at 48 and 72 h after DH running (*p* = 0.003 and *r* = 0.61 for POST-48H; *p* = 0.006 and *r* = 0.56 for POST-72H). The GPRS value was significantly increased after DH running after the supplementation of both P and AG, but no obvious difference was observed.

### 3.2. Lipid Peroxidation

**Figure 3:**
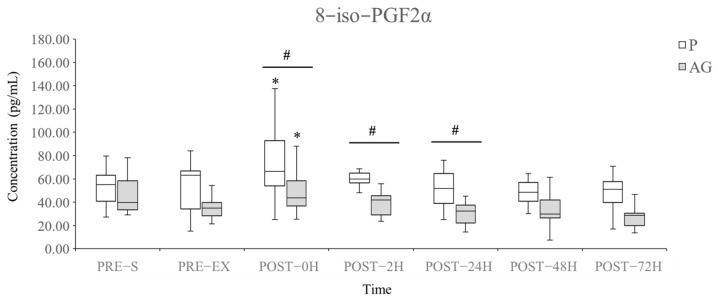
Levels of plasma 8-iso-PGF2α during the experimental period. Q1, median, Q3, minimum, and maximum values are presented in boxplots (*N* = 12). * *p* < 0.05 vs. PRE-EX; # *p* < 0.05 between P and AG supplementation. AG: American ginseng; P: placebo; 8-iso-PGF2α: 8-iso-prostaglandin F 2α.

The level of plasma 8-iso-PGF2α was used to evaluate lipid peroxidation following DH running. As shown in Figure 3, AG supplementation reduced the plasma 8-iso-PGF2α levels compared with P supplementation immediately (*p* = 0.01 and *r* = 0.53), 2 h (*p* = 0.01 and *r* = 0.53), and 24 h (*p* = 0.028 and *r* = 0.45) after DH running. However, short-term supplementation of AG did not affect the basal level of plasma 8-iso-PGF2α before DH running (P vs. AG at PRE-S, *p* = 0.114 and *r* = 0.32; P vs. AG at PRE-EX, *p* = 0.112 and *r* = 0.32). Both P and AG supplementation significantly elevated the plasma 8-iso-PGF2α levels following DH running (PRE-EX vs. POST-EX in P: +57.73%, *p* = 0.034 and *r* = 0.43; PRE-EX vs. POST-EX in AG: +37.56%, *p* = 0.019 and *r* = 0.48). AG supplementation led to the inhibition of plasma 8-iso-PGF2α production within 24 h after DH running.

### 3.3. Inflammatory Cytokines

**Figure 4:**
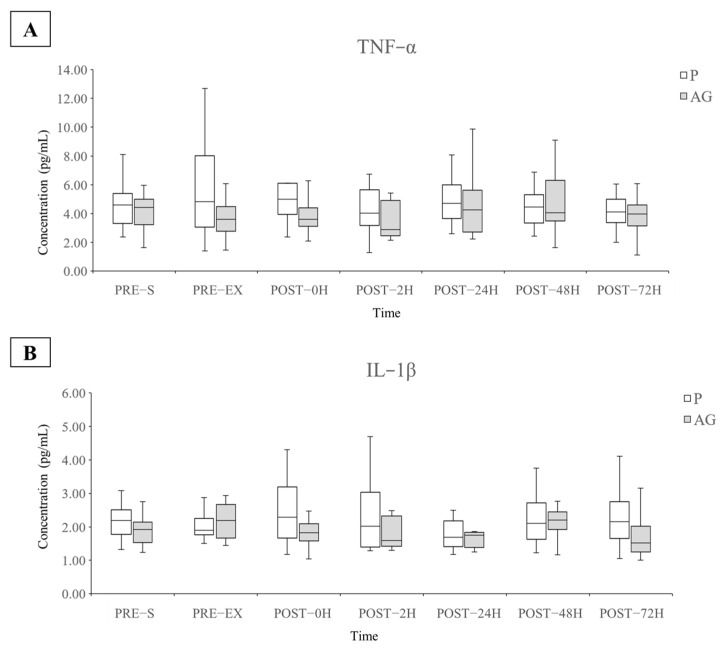
Levels of plasma TNF-α and IL-1β during the experimental period (*N* = 12). Q1, median, Q3, minimum, and maximum values of TNF-α (**A**) and IL-1β (**B**) are presented in boxplots. AG: American ginseng; IL-1β: interleukin-1β; P: placebo; TNF-α: tumor necrosis factor-α.

**Figure 5:**
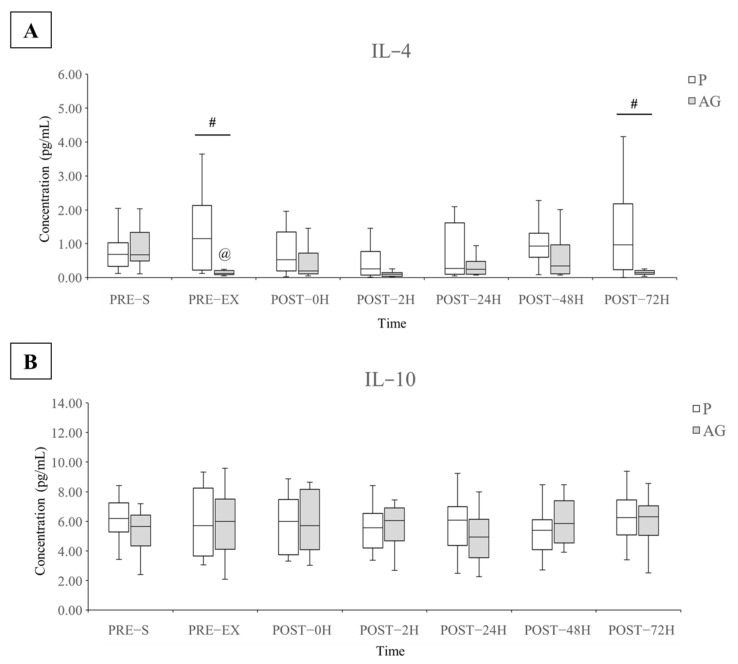
Levels of plasma IL-4 and IL-10 during the experimental period (*N* = 12). Q1, median, Q3, minimum, and maximum values of IL-4 (**A**) and IL-10 (**B**) are presented in boxplots. @ *p* < 0.05 vs. PRE-EX; # *p* < 0.05 between P and AG supplementation. AG: American ginseng; IL-4: interleukin-4; IL-10: interleukin-10; P: placebo.

The plasma TNF-α, IL-1β, IL-4, and IL-10 cytokine levels were used to evaluate the pro- and anti-inflammatory responses before and after supplementation and DH running. A significant difference in the plasma IL-4 levels was observed between the P and AG supplementation, whereas no alteration was found in the levels of other inflammatory cytokines (Figure 4 and Figure 5). As shown in Figure 5, short-term AG supplementation significantly reduced the resting plasma IL-4 levels before and 72 h after DH running (*p* = 0.002 and *r* = 0.52 for PRE-EX; *p* = 0.028 and *r* = 0.45 for POST-72H). Moreover, the AG supplementation led to a slight but not significant increase in the plasma IL-4 levels immediately (*p* = 0.099 and *r* = 0.34) and 48 h (*p* = 0.075 and *r* = 0.36) after DH running.

## 4. Discussion

In this study, we have demonstrated that a 30-day supplementation of AG attenuated DH running induced oxidative stress and muscle damage in healthy male subjects. Our main findings were that AG supplementation suppressed the increased production of plasma 8-iso-PGF2α levels during DH running and resulted in a greater recovery rate of plasma CK activity from day 2 to day 3 after DH running but did not affect muscle soreness. Moreover, the resting plasma IL-4 levels were significantly reduced after AG supplementation, whereas the levels of all the inflammatory cytokines remained unchanged by DH running.

Ginsenosides, especially Rg1, Rb1, and Re ginsenosides, are critical bioactive components in ginseng extract that have been extensively investigated because of their antioxidant and anti-inflammatory properties. Therefore, ginsenoside content plays a crucial role in the therapeutic effects of ginseng. In rodent studies, the oral dosage ranges from 10 to 60 mg/kg/day have been found to be safe and effective against oxidative stress and inflammation [28,29,30,31]. According to the practical guidance for converting animal doses to humans sourced from the U. S. Food and Drug Administration [32], the human equivalent doses are calculated by dividing the doses of rats and mice by conversion coefficients 6.2 and 12.3, respectively. The effective doses for laboratory animals were transformed into those for humans, and the effective dose corresponding to the average body weight (70.2 kg) in this current study ranged from 56.6 to 679.3 mg of ginsenosides daily. The percentage of total ginsenosides contained in AG capsules is 15.7% (*w*/*w*); hence, the actual dose of ingested ginsenosides for each subject was 252.2 mg/day. This applicable dose is within the effective range required for ginsenoside treatment. In addition, our previous study has shown that AG supplementation exhibits anti-fatigue potential [17].

The bioavailability of most ginsenosides is poor. In rodent studies, the bioavailability of ginsenosides Rg1, Rb1, and Re were found to range from 6.06 to 18.4%, 1.18 to 4.35%, and 0.28 to 7.06, respectively [33,34,35]. However, a part of the ingested ginsenosides is transformed into ginsenoside metabolites by the gut microbiome. Protopanaxadiol (PPD), protopanaxatriol (PPT), and compound K, which possess high bioactivity, are important metabolites that have attracted considerable attention [36]. Regarding the ginsenosides used in this study, Rb1, Rc, and Rd can be converted to compound K and PPD, and Re can be converted to PPT after ingestion [36,37]. However, ginsenosides and their metabolites have a long half-life in systematic circulation, ranging from 10 to 20 h [36,38]. According to the accumulation effects of PPD-type ginsenosides [39], we consider that the AG supplementation reached an effective dose to regulate the physiological stress induced by DH running under this specific duration and dosage of supplementation.

Similar to those of previous studies in rats and humans [17,40,41], our results showed that the short-term continuous supplementation of ginseng extract may induce an increased recovery rate of plasma CK activity following DH running. Although significant differences were not observed between P and AG supplementation at all time points in the current study, AG supplementation showed a better recovery pattern at 48 h and 72 h post DH running. Damage to the muscle fiber structure, lipid peroxidation of cell membranes, and/or inflammatory reactions are involved in promoting the efflux of CK into the circulation [41,42]. The influence of AG extract on plasma CK activity may be attributable to the decrease in the production of plasma 8-iso-PGF2α and its related oxidative damage following DH running. Although DH running resulted in a significant increase in plasma CK activity, our results revealed a smaller response in plasma CK activity following DH running compared to that observed in previous studies [4]. The subject’s physical conditioning, training experience, slope setting of the treadmill, exercise intensity, and exercise duration may affect the response to muscle damage following eccentric exercise. In our study, a shorter interval between pretest (i.e., determination of $\dot{V}O_{2}$peak and confirmation of exercise intensity of DH running) and DH running might have resulted in the repeated bout effect and subsequently weakened the magnitude of muscle damage [43,44]. AG supplementation did not improve muscle soreness during the recovery period after DH running. This finding can be explained by a previous investigation, which suggests that muscle soreness is not an appropriate indicator for the determination of EIMD and inflammation [45].

In our study, AG supplementation downregulated lipid peroxidation, as determined by the plasma 8-iso-PGF2α concentration observed following DH running. Our results are consistent with those of previous studies, which showed that an increase in the products of lipid peroxidation is found following eccentric exercise [7,46]. Evidence from a rat study revealed that DH running causes obvious oxidative stress in contracting muscles because of increased levels of xanthine oxidase activity, protein carbonyl, and uric acid [47,48]. It is noteworthy that the xanthine oxidase-related mechanism plays a critical role in the production of reactive oxygen species (ROS) and the suppression of Ca^2+^ ATPase activity. Our results agree with those findings of a previous study, which reported that moderate ginseng supplementation attenuates exhaustive exercise induced lipid peroxidation [49]. Similarly, a 28-day ginsenoside Rb1 supplementation resulted in a decrease in the levels or activities of serum CK and LDH and liver malondialdehyde (MDA) and antioxidant enzymes in mice after exhaustive swimming [40]. Ginsenosides exert a protective effect on the membrane-stabilizing capacity against the increase in the production of lipid and protein peroxidation induced by acute exercise, which has been suggested as a possible mechanism [48,50,51,52]. Alternatively, a 3-month administration of ginseng extract displayed similar protective effects on isoproterenol-induced myocardial injury, which was contributed by promoting the activities of antioxidant enzymes and attenuating lipid peroxidation [52]. Taken together, in agreement with previous findings from those mentioned studies, the continuous supplementation of ginseng extract may improve endogenous antioxidant systems against EIMD-induced ROS generation.

In this study, no marked influence on plasma pro-inflammatory cytokines TNF-α and IL-1β following DH running was observed. Numerous studies have reported that eccentric exercise promotes inflammatory reactions by mediating leukocyte function and inflammatory cytokine secretion [46,53,54,55,56,57]. Exceedingly high or chronic expression of pro-inflammatory cytokines may contribute to the ROS generation capability of leukocytes or the impairment of muscle regeneration and repair, resulting in longer processes for returning the pre-exercise performance [58]. However, findings similar to our results have been reported [59,60,61] in studies that revealed no impact of DH running or other eccentric exercises on the inflammatory response, even with DOMS, decreased maximum voluntary contraction force, and/or increased CK activity.

As mentioned in this manuscript, IL-6 is the most frequently changed inflammatory cytokine following eccentric exercise with low to moderate intensity and duration [53,56]. A review article suggested that there is no significant association between local and systemic cytokine responses to exercise besides IL-6 [62]. Intramuscular IL-6 gene expression and protein concentration are markedly upregulated, and IL-6 is released into the circulation during prolonged knee-extensor exercise, but no changes in response to intramuscular and circulating TNF-α have been observed [63]. Another rodent study was conducted to investigate how endotoxin lipopolysaccharide (LPS)-induced inflammatory reactions mediate the production and secretion of intramuscular cytokines [64]. The results revealed that, although skeletal muscle contributes to an important source of cytokine production, it displays significant differences in the levels of TNF-α and IL-1β between muscle interstitial fluid and plasma. In addition, another explanation regarding this phenomenon suggests that some plasma cytokines are rapidly excreted into urine; thus, the total number of cytokines produced in the body cannot be precisely understood by the measurement of plasma cytokines [65].

In addition to measuring pro-inflammatory cytokines, the measurement of plasma IL-4 and IL-10 was presented as the response of anti-inflammatory cytokines following DH running. Our results showed that the resting concentration of plasma IL-4 was attenuated by AG supplementation before and 72 h after DH running. In contrast, there was a trend toward an increase in plasma IL-4 immediately and 48 h after DH running after AG supplementation instead of P supplementation. These important findings indicated that ginseng supplementation may modulate the resting and post-DH running plasma IL-4 levels in human subjects. In several inflammatory models manipulated by chemicals or diseases, ginseng extract and/or ginsenosides improve severe inflammatory reactions and allergic symptoms by suppressing the levels of pro-inflammatory cytokines [66,67]. IL-4 acts as an anti-inflammatory factor by attenuating the apoptotic activities of neutrophils while promoting the inflammation resolution of macrophages [68]. In addition to its anti-inflammatory effects, IL-4 has been suggested to have a novel regulatory role in the processes of muscular adaptations to training or exercise [69]. In both young and elderly males, intramuscular IL-4 protein expression tends to decrease following acute exercise in untrained individuals, whereas the 12-week resistance training alters the previous response by the increased intramuscular IL-4 protein expression following acute exercise. This finding could be explained by previous studies [70,71], which suggest that IL-4 and its IL-4α receptors play an important role in controlling myogenesis. Collectively, AG may improve inflammatory adaptation during muscular regeneration following DH running by modulating IL-4 production and secretion. In contrast to that of IL-4, the regulatory role of IL-10 has been increasingly studied in anti-inflammatory effects following exercise. However, the plasma IL-10 levels were not altered by AG supplementation or DH running. A previous study showed that DH running led to a significant increase in the level of plasma myoglobin but did not influence the plasma levels of IL-10 and IL-6 [65]. The results suggest that exercise intensity is more critical than muscle damage in controlling the systemic release of IL-10 and IL-6.

We agree that antioxidant supplementation may help athletes improve the capacity of the antioxidant defense system following daily training [5]. On the other hand, eccentric exercise has been shown to promote varied muscle adaptations. However, a previous animal study reported that chronic eccentric exercise promoted endogenous antioxidant enzymes and mitochondrial function but did not reduce overall oxidative stress [72]. Thus, the appropriate use of anti-inflammatory and antioxidant substances will contribute to better recovery following EIMD. Although this study did not evaluate any physical performance or muscular function, our results provided partial evidence that short-term AG supplementation may relieve eccentric EIMD.

Although our main findings suggest that AG supplementation reduces oxidative stress and enhances inflammatory adaptation following DH running in human subjects, there are notable limitations to this study. The main limitation is that some of the major findings with insufficient statistical power are due to the small sample size. A post hoc power analysis showed that sufficient statistical power was given to detect significant differences of plasma 8-iso-PGF2α levels between supplementations (87% for POST-0H, 85% for POST-2H, and 59% for POST-24H). Whereas, insufficient statistical power was found when AG supplementation induced the significant change in plasma IL-4 concentration (65% for PRE-EX and 55% for POST-72H). Moreover, the EIMD model used in this study did not significantly alter the levels of circulating inflammatory cytokines as expected. These two factors may result in the impacts of AG supplementation on circulating inflammatory cytokines not being able to be accurately detected before and after DH running. Thus, further investigations with the appropriate sample size and the modified DH running protocols or other EIMD models are needed to examine the effects of AG supplementation on the exercise-induced inflammatory response.

## 5. Conclusions

In summary, our study suggests that a short-term AG supplementation alleviates EIMD caused by DH running in males. This protective effect of AG on EIMD is probably contributed by the decrease in lipid peroxidation during exercise and the promotion of inflammatory adaptation. However, the experimental muscle injury model applied in this study may merely induce a lower degree of muscle damage due to the repeated bout effect or insufficient stimulation. Hence, further investigation is required to examine the different potential protective mechanisms and clinical potential of AG on EIMD.
